# *Panax notoginseng* Saponins for Treating Coronary Artery Disease: A Functional and Mechanistic Overview

**DOI:** 10.3389/fphar.2017.00702

**Published:** 2017-10-17

**Authors:** Lian Duan, Xingjiang Xiong, Junyuan Hu, Yongmei Liu, Jun Li, Jie Wang

**Affiliations:** ^1^Department of Cardiology, Guang'anmen Hospital, China Academy of Chinese Medical Science, Beijing, China; ^2^Graduate School, Beijing University of Traditional Chinese Medicine, Beijing, China

**Keywords:** *Panax notoginseng*, PNS, coronary artery disease, traditional Chinese medicine, review

## Abstract

Coronary artery disease (CAD) is a major public health problem and the chief cause of morbidity and mortality worldwide. *Panax notoginseng*, a valuable herb in traditional Chinese medicine (TCM) with obvious efficacy and favorable safety, shows a great promise as a novel option for CAD and is increasingly recognized clinically. Firstly, this review introduced recent clinical trials on treatment with PNS either alone or in combination with conventional drugs as novel treatment strategies. Then we discussed the mechanisms of *P. notoginseng* and *Panax notoginseng* saponins (PNS), which can regulate signaling pathways associated with inflammation, lipid metabolism, the coagulation system, apoptosis, angiogenesis, atherosclerosis, and myocardial ischaemia.

## Introduction

Coronary artery disease (CAD) is a major public health problem and a chief cause of morbidity and mortality worldwide. The number of deaths due to CAD was 56 million people globally during a decade from 2000 (WHO, 2014). The sum of hospitalized cardiovascular operations rised by 28%, from about 6 to 7.5 million (Mozaffarian et al., 2016). The economic load of caring for patients also represents a huge cost for society. Revascularization represents innovative progress in the treatment for symptomatic CAD. However, by targeting one or two vascular lesions, it fails to completely solve the problem of plaque progress. A truly advance in the treatment of CAD will require more effective prevention. At the end of the nineteenth century, drug resistance to organic nitrates was observed (Dilidar, 2009; Münzel et al., 2011), while aspirin resistance was observed in 1994 (Helgason et al., 1994). Clopidogrel has been widely used in various thrombotic diseases, especially in CAD patients with percutaneous coronary intervention (PCI; O'Gara et al., 2013). About a quarter of patients administering standard loading of clopidogrel exhibit poor responsiveness (Serebruany et al., 2005). Due to the complexity of CAD, most patients require lifelong medication. Moreover, oral aspirin may directly stimulate the gastric mucosa and initiate abdominal discomfort, nausea and vomiting. The long-term use of aspirin can easily cause gastric mucosal damage (Hirsh et al., 1995). The risk of diabetes greatly increases with large doses of statins. In addition, statins can cause abnormal liver enzymes and myopathy (Thompson et al., 2016). Varying degrees of drug resistance and adverse reactions increase the difficulty and dissatisfaction with treatment.

In recent years, Traditional Chinese Medicine (TCM) has gained widespread popularity. Furthermore, an increasing number of studies have confirmed the efficacy of TCM for treating CAD. In 2007, nearly 4 out of 10 adults had used TCM therapy in the past 12 months, with natural products as the most commonly used therapies (Barnes et al., 2008). Of the various natural products, *Panax notoginseng* is one of the most commonly applied products because it has been evaluated with various beneficial effects, such as promotion of blood circulation, cerebrovascular protection (Liu L. et al., 2014; Zhang et al., 2016), improvement of neurological function (Gao et al., 2012), reduction of oxidative stress (Ding et al., 2015), and mitigation of bone loss (Fan et al., 2015).

*Panax notoginseng* is particularly popular among patients with CAD because many studies have been associated the consumption of *P. notoginseng* with CAD treatment. *Panax notoginseng* saponin (PNS) is the main active ingredient of *P. notoginseng*. Over the past over 40 years, great energy has been devoted to confirming the effectiveness of the compounds of *P. notoginseng* on CAD. Many animal experiments have shown that PNS can improve the energy metabolism of myocardial cells, reduce myocardial damage, and inhibit ischaemia-induced cardiomyocyte apoptosis in acute MI rats (Chen et al., 2011; Yue et al., 2012). Now the compound of PNS is available as an over-the-counter drug in both China and worldwide. In China alone, 5,000 tons of *P. notoginseng* products are produced annually (Liu J. et al., 2014). The role of *P. notoginseng* in cardiovascular diseases has been summarized (Liu J. et al., 2014; Yang et al., 2014), but no articles have focused on the effects of *P. notoginseng* against CAD. This review summarizes extensively recent evidence on the use of *P. notoginseng* in CAD therapy, its therapeutic effects and adverse events. Our current understanding of *P. notoginseng* cardioprotective effects and mechanisms against CAD will also be discussed in detail.

## Methodology

The terms “*P. notoginseng*” or “Sanqi” or “*P. notoginseng* saponins” were searched as “Title/Abstract” and MeSH terms in PubMed, China National Knowledge Infrastructure (CNKI) and SinoMed database. Articles related to therapeutic effects in coronary artery disease (CAD) were picked out manually. All articles with abstract were included and no language restrictions was applied.

## Biological characteristics of *Panax notoginseng*

### Brief history

**Figure 1:**
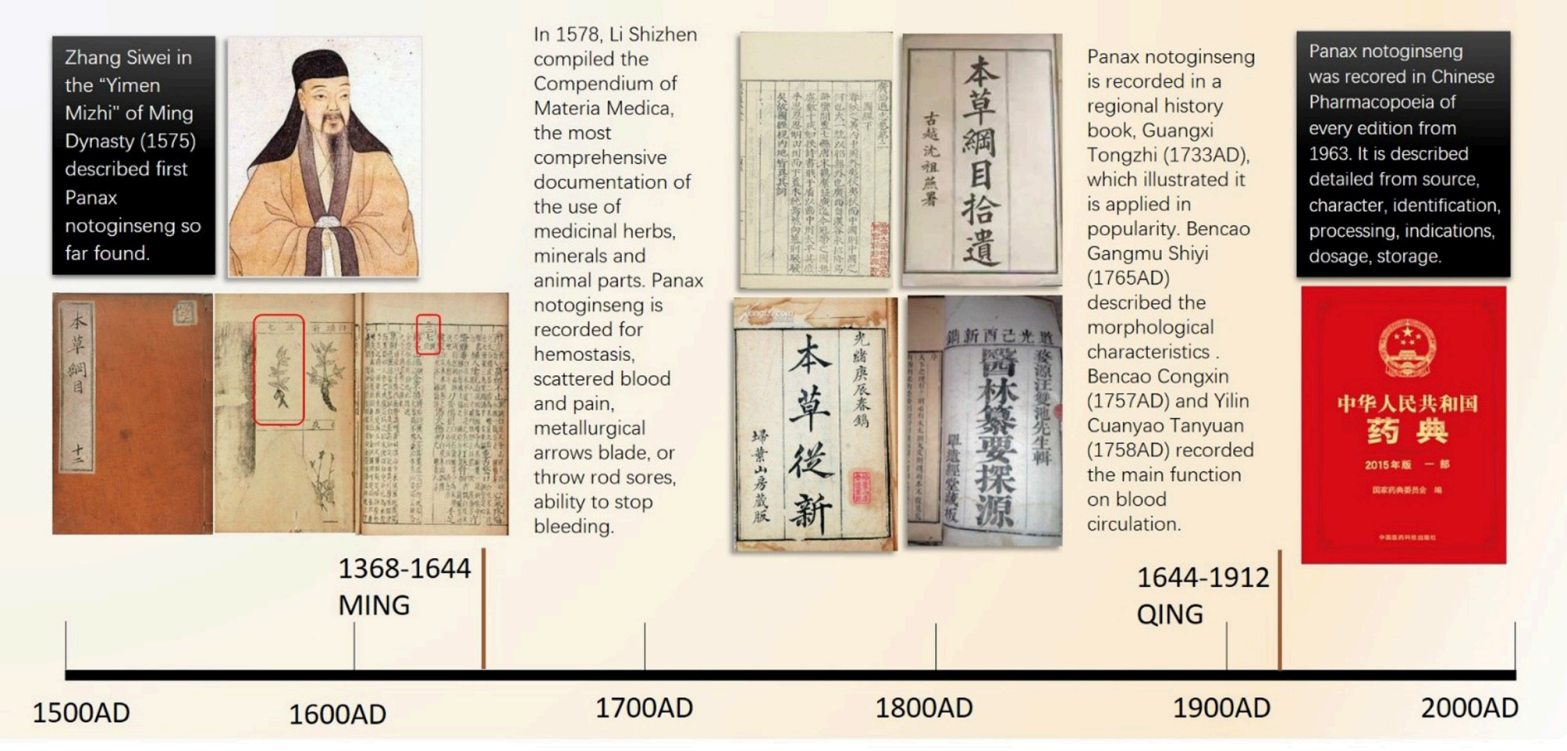
The important person and classic medical books in which *Panax notoginseng* was recorded. Zhang Siwei recorded first *Panax notoginseng*. The compendium of Materia Medica described the function of *Panax notogingseng* in detail. And then *Panax notoginseng* is captured in four significant ancient medical books and Chinese Pharmacopoeia.

*Panax notoginseng* is a medicinal plant that was first used by ethnic minorities in China. It has been one of the most acclaimed herbs in China for 400 years. *Panax notoginseng* is traditionally applied as an anodyne and a hemorheologic-altering drug. The main medical component is the radix of *P. notoginseng*, also known as Sanqi, Tianqi, and Shanqi in East Asian countries (Wang T. et al., 2016). “Compendium of Materia Medica” (Bencao Gangmu 本草纲目) recorded the official detailed medical applications of *P. notoginseng* in 1758, in which *P. notoginseng* is called “more precious than gold” (jinbuhuan 金不换) and written as “三七 ” in Chinese (red box in Figure 1). As one species of ginseng, it was unrecognized worldwide until American ginseng was discovered in 1716. In 1843, the Russian botanist Carl Meyer nominate Asian ginseng the botanical name “Panax,” which implies “all-healing” in Greek (Wong et al., 2015). “Noto” means “back, spine,” and “ginseng” represents “essence of men.”

### Botanical characteristics

**Figure 2:**
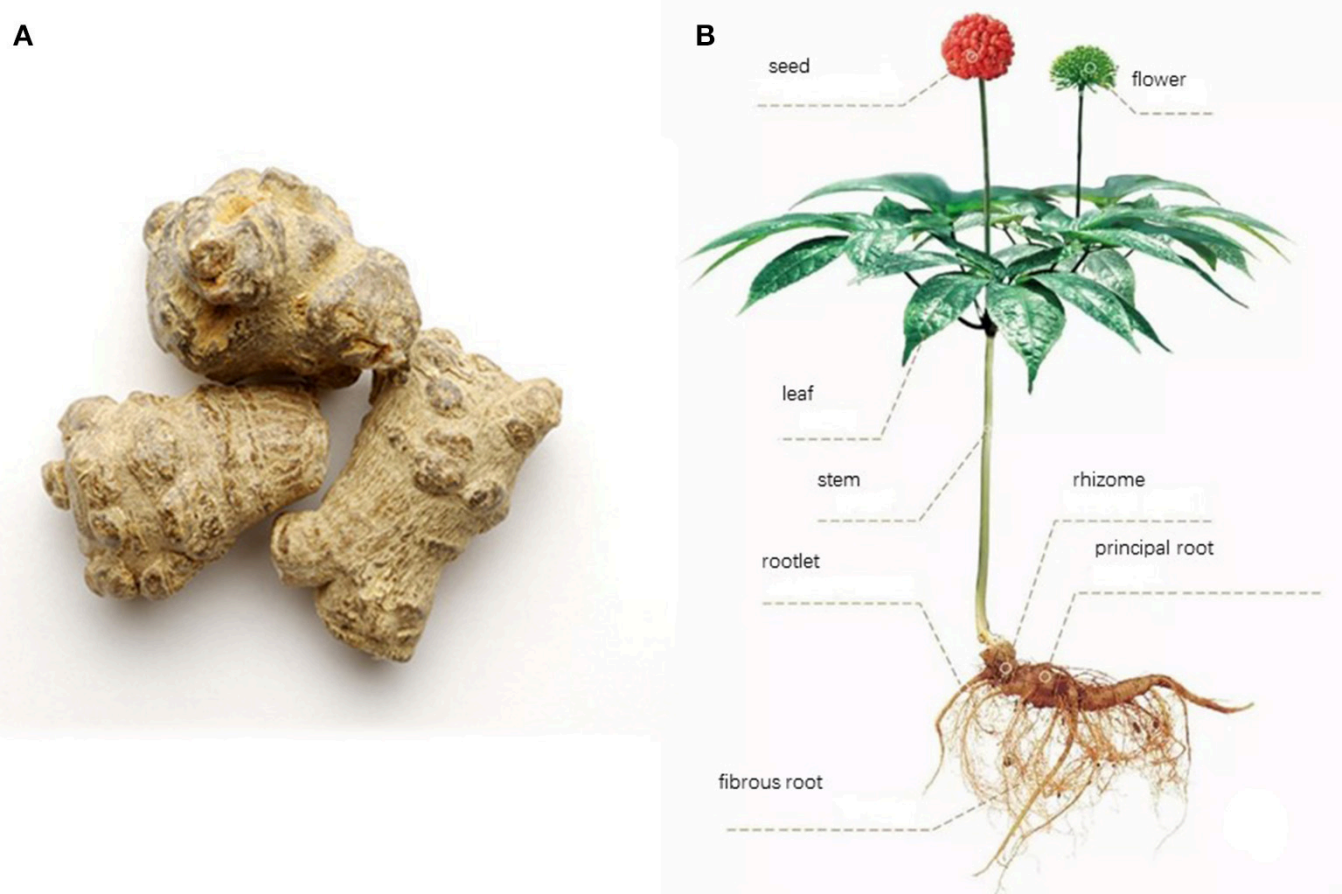
Radix **(A)** and plants pictures **(B)** for *Panax notoginseng* F.H. Chen. The whole plant looks like **(B)**. The main part used for medical purpose is the principal root which looks like **(A)** after cleaning and pruning.

*Panax notoginseng* F.H. Chen is a hemitropous perennial herb with a short rhizome, a bamboo whip, and 2 to several fleshy roots (Editorial Board of Flora of China, 1978). The taproot looks conical or cylindrical with a length of 1–6 cm and a diameter of 1–4 cm. The surface is grayish brown or grayish yellow with intermittent vertical wrinkles and branch marks. There are stem scars on top with surrounding tumor-like bulges. The characteristics include dense, solid gray green, yellow green, or gray sections (Figure 2).

### PNS: the major therapeutic constituents of *Panax notoginseng*

**Figure 3:**
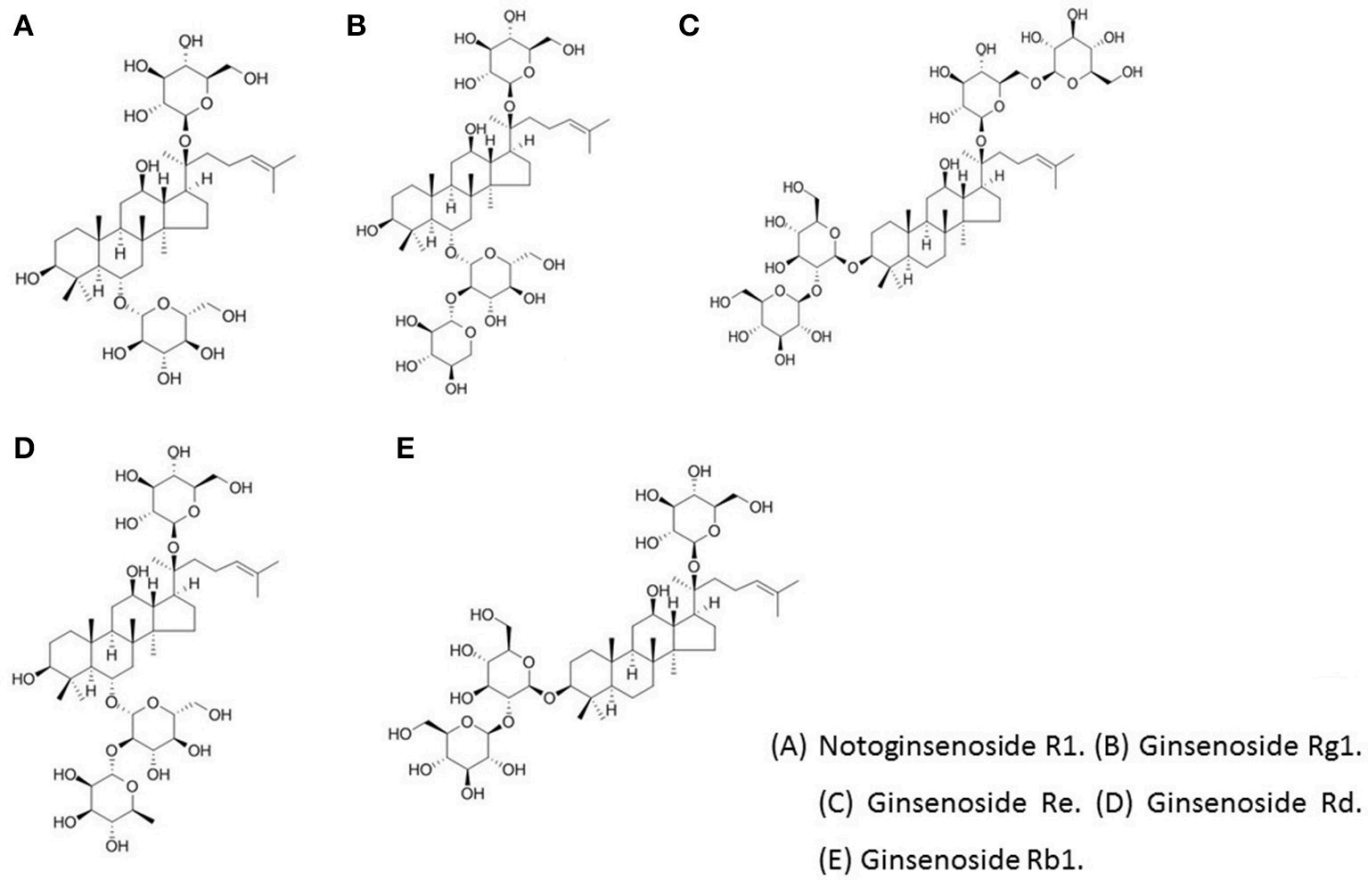
The chemical structure of the main active ingredients of PNS. **(A)** Notoginsenoside R1. **(B)** Ginsenoside Rg1. **(C)** Ginsenoside Re. **(D)** Ginsenoside Rd. **(E)** Ginsenoside Rb1^1^.

*Panax notoginseng* contains three key constituents, including saponins, polysaccharides, and dencichine (Kim, 2012). Polysaccharides have many physiological functions, such as anti-tumor and immune regulation activity (Gu et al., 2015). A special type of amino acid, dencichine, is an active substance of *P. notoginseng*. Its structure is β-NoXalo-L-α,β diaminopropionic acid and can be artificially synthetized (Wei and Wang, 1987). Saponins constitute one of the most important effective components of *P. notoginseng*. So far, more than 80 kinds of monomer saponins have been isolated from *P. notoginseng* parts (radix, stem, leaf, alabastrum, seed, etc.) since their first separation and identification in 1979 (Wu, 1979). However, the most important components of PNS are ginsenoside Rg1, Rb1, Re, and notoginsenoside R1 (NR1; Figure 3). The amounts of these four components in PNS are 30, 2.5, 20, and 2.5%, respectively. The radix of *P. notoginseng* is the main part for extraction. According to the different aglycone structures, saponins are divided into two categories: dammarane tetracyclic triterpene and oleanane type pentacyclic triterpene. Based on whether the mother molecular nucleus structure of C6 displaces a hydroxyl group, dammarane-type saponins can be divided into protopanaxadiol (PPD), protopanaxatriol (PPT), or octotillol (Wong et al., 2015). Ginsenoside Rb1 is one of the major protopanaxadiol-type saponins. Ginsenoside Re, Rg1, and NR1 are the main raw ginseng triol saponins (Wong et al., 2015).

### Quality control

Many plant species are named Sanqi (*P. notoginseng*). According to the statistics of the reference literature, except for Araliaceae *P. notoginseng*, medicinal plants named Sanqi (*P. notoginseng*) include as many as 20 species belonging to 11 families, which causes difficulty in distinguishing real *P. notoginseng*. Pharmacological studies indicated that ginsenoside Rg1, ginsenoside Rb1, and NR1 are the main active constituents of *P. notoginseng*. The quality control of *P. notoginseng* also focuses on these three components. According to the “Chinese Pharmacopoeia,” the distinction process is as follows: precisely extract the control solution and the test solution, pour into a liquid chromatograph, and determine the species. The total amounts of ginsenoside Rg1 (C42H72O14), ginsenoside Rb1 (C54H92O23), and NR1 (C47H80O18) should not be <5%. In standard Chinese Medicinal Materials in Hongkong (HKCMMS, 2017), *P. notoginseng* can be identified by thin layer chromatography. The chromatographic results indicate that the Rb1, Rg1, and NR1 have the same colors and Rf values. In addition, the relative retention time of *P. notoginseng* and the characteristic peaks of the six extraction liquids should be in accordance with the standard by HPLC fingerprint identification.

## The therapeutic effects of PNS on CAD

### Search strategy

We conducted a systematic search of oral PNS for over 4 weeks against CAD on four English databases and four Chinese databases: MEDLINE, the Cochrane Central Register of Controlled Trials (CENTRAL), EMBASE Database, WHO Clinical Trials Registration Platform, Chinese National Knowledge Infrastructure (CNKI), Chinese Scientific Journal Database (VIP), WANFANG, and SinoMed. The search time frame ranged from the databases' inception until 20 Feb 2017. We also searched reference lists for further publications. The search expression used in MEDLINE was ((“coronary heart disease” [MeSH Terms] OR (“coronary artery disease” [MeSH Terms] AND (“*P. notoginseng*” [MeSH Terms] OR sanqi[Text Word] OR sanchi[Text Word])) OR Xuesaitong[Text Word] OR Xueshuantong[Text Word])) AND “Randomized Controlled Trial” [Publication Type:NoExp]. Similar expressions were used in the other databases. Outcome measures meet the primary or secondary outcomes.

### Study quality

Seventeen randomized clinical trials with 1,747 participants was collected which randomly assigned to a conventional treatment vs. a PN preparation evaluated cardiovascular outcomes (Table 1). The quality of the 17 RCTs was evaluated from seven aspects using the ROB scale in the Cochrane handbook (Table 2). Three RCTs indicated the random way as random numbers. However, the other studies didn't describe the random method. Two RCT (Han, 2008; Teng, 2014) refers to random concealment and blindness.

**Table 1:** The basic information of the 17 RCTs of PNS on CAD.

**Study ID**	**Sample(T/C)**	**Age, Mean ± SD T,y**	**Age, Mean ± SD C,y**	**M/F T**	**M/F C**	**Intervention group**	**Course (weeks)**	**Control group**	**Outcome measures**	**References**
Du, 2009	56/56	58.8 ± 9.2	58.8 ± 9.2	Unclear	Unclear	XST + conventional drugs	4	Conventional drugs	FAA, DAA, DN	Du and Chen, 2009
Feng, 2016	36/35	69.3 ± 4.8	69.4 ± 5.2	21/15	20/15	PNS	12	Atorvastatin	lipid, PEP	Feng et al., 2016
Han, 2008	30/30	64.1 ± 10.8	63.7 ± 11.7	23/7	21/9	XST + conventional drugs	12	Conventional drugs	FAA, DAA	Han, 2008
Hou, 2016	42/42	62.3 ± 2.31	62.4 ± 2.32	23/19	22/20	XST + conventional drugs	4	Conventional drugs	FAA, DAA, ECG	Hou, 2016
Kong, 2006	52/52	61.2 ± 5.73	60.77 ± 5.61	31/21	32/20	XST + conventional drugs	4	Conventional drugs	FAA, ECG	Kong and Zhang, 2006
Kuang, 2011	90/90	56.3 ± 6.9	57.1 ± 7.2	47/43	46/44	XST + conventional drugs	4	Conventional drugs	FAA, DAA	Kuang et al., 2011
Liu, 2008	30/30	64.6 ± 5.4	63.6 ± 4.5	Unclear	Unclear	XST + conventional drugs	4	Conventional drugs	ECG, lipid	Liu et al., 2008
Meng, 2013	600/600	68 ± 11	69 ± 9	421/179	368/232	PNS tablet + conventional drugs	52	Conventional drugs	PEP	Meng et al., 2013
Song, 2005	50/50	61.2 ± 5.73	60.8 ± 5.61	31/19	33/17	XST + conventional drugs	4	Conventional drugs	FAA, DN, ECG	Song et al., 2005
Teng, 2014	40/40	70.7 ± 6.87	71.7 ± 4.32	17/23	21/19	XST + conventional drugs	4	Conventional drugs+XST capsule placebo	FAA, lipid	Teng, 2014
Wan, 2011	26/26	65.7	Unclear	15/11	13/13	XST + conventional drugs	4	Conventional drugs	ECG	Wan, 2011
Wei, 2010	90/90	60.4 ± 3.5	60.4 ± 3.5	Unclear	Unclear	XST +conventional drugs	4	Conventional drugs	FAA, DAA	Wei, 2010
Yan, 2015	28/27	76.3 ± 9.04	76.32 ± 9.04	Unclear	Unclear	Sanqi Tongshu capsule +aspirin	24	Aspirin	PEP	Yan et al., 2015
Yu, 2010	50/50	64.2 ± 12.13	62.8 ± 10.8	29/21	28/22	XST + conventional drugs	4	Conventional drugs	ECG	Yu, 2010
Zhang, 2014	30/30	60 ± 3.4	61 ± 4.0	16/14	15/15	XST + trimetazidine	4	Trimetazidine	FAA, DAA	Zhang, 2014
Zheng, 2014	56/56	Unclear	Unclear	Unclear	Unclear	XST + conventional drugs	4	Conventional drugs	ECG	Zheng, 2014
Zhou, 2009	43/43	65 ± 6	65 ± 6	32/11	34/9	XST + conventional drugs	4	Conventional drugs	ECG	Zhou and Bai, 2009

*T/C, Treatment group/control group; M/F, Male/female; XST, Xuesaitong soft capsule; FAA, frequency of angina attack; DAA, duration of angina attack; DN, dosage of nitroglycerin; PEP, the primary end point*.

**Table 2:** Risk of bias in the 17 RCTs of PNS on CAD.

**References**	**A**	**B**	**C**	**D**	**E**	**F**	**G**
Du and Chen, 2009	?	?	?	?	?	?	?
Feng et al., 2016	?	?	?	?	?	?	?
Han, 2008	+	+	+	+			
Hou, 2016	?	?	?	?	?	?	?
Kong and Zhang, 2006	?	?	?	?	?	?	?
Kuang et al., 2011	?	?	?	?	?	?	?
Liu et al., 2008	+	+	+	?	?	?	?
Meng et al., 2013	+	?	?	?	?	?	?
Song et al., 2005	?	?	?	?	?	?	?
Teng, 2014	+	+	+	+	?	?	?
Wan, 2011	?	?	?	?	?	?	?
Wei, 2010	?	?	?	?	?	?	?
Yan et al., 2015	?	?	?	?	?	?	?
Yu, 2010	?	?	?	?	?	?	?
Zhang, 2014	?	?	?	?	?	?	?
Zheng, 2014	?	?	?	?	?	?	?
Zhou and Bai, 2009	?	?	?	?	?	?	?

*A, Random sequence generation; B, allocation concealment; C, blinding of participants and personnel; D, blinding for outcome assessment; E, incomplete outcome data; F, selective reporting; G, others bias; +, low risk of bias; −, high risk of bias; ?, unclear risk of bias*.

### Primary outcome

The primary outcome of CAD is the primary end point which was defined as the composite of all-cause mortality, myocardial infarction (MI), revascularization, and rehospitalization for unstable angina. PNS has been observed to have several beneficial effects in patients with different stages of CAD. Several RCTs reported oral PNS could reduce the primary outcome. In 2008, a team underwent a RCT of 60 patients with CAD after PCI. The patients who had PNS (120 mg every time, three times every day) or a placebo was prescribed daily in combination with their conventional therapy for 3 months. The end point, rehospitalization, was focused on. The rehospitalization rate of patients with PNS was better than in the control group (1/30 and 3/30; Han, 2008) (Table 3). In 2013, furthermore, another team conducted a 1-year RCT with ~1,200 CAD patents, 600 patients in the experimental group were given PNS (300 mg every time, three times every day). PNS increased the inhibitory effect of clopidogrel on platelet aggregation and reduced the primary end points. This trial compared the incidence of the primary end points in the experiment and control groups, which was 3.3% (20 cases) and 7.8% (47 cases; Meng et al., 2013), resepctively. The primary end points included cardiac death, myocardial infarction, revascularization, stent thrombosis, of which most were related to revascularization.

**Table 3:** The end point with PNS+conventional drugs and conventional drugs alone.

		**PNS+conventional drugs**	**Conventional drugs**	***P*-value**
Cardiac death	52 w	1/600	1/600	>0.05
	12 w	0/30	0/30	>0.05
Myocardial infarction	52 w	2/600	4/600	< 0.05
	12 w	0/30	0/30	>0.05
Revascularization	52 w	16/600	37/600	<0.05
	12 w	0/30	0/30	>0.05
Stent thrombosis	52 w	1/600	5/600	<0.05
Rehospitalization for unstable angina	12 w	1/30	3/30	>0.05

### Secondary outcomes

Secondary outcomes include electrocardiogram (ECG), attack of angina pectoris, such as frequency of angina pectoris, duration of angina pectoris and dosage of nitroglycerin, quality of life. Two systematic reviews estimated current evidence for the benefit of secondary outcomes and adverse events of PNS for CAD. One systematic review included 17 randomized clinical trials. Oral PN could alleviate angina pectoris (Shang et al., 2013). Another systematic review including a total of six RCTs with 716 participants on unstable angina pectoris(UA) studied PNS alone or combined with conventional drugs vs. conventional drugs alone. The results illustrated that PNS combined with conventional drugs displayed also a significant effect on relieving angina symptoms and improving ECG compared with conventional drugs alone (Yang et al., 2013).

#### Attack of angina pectoris

Angina pectoris is the symptoms for chest pain or discomfort due to CAD (Xiong et al., 2015). The patients may also feel the discomfort in your neck, jaw, shoulder, back, or arm. Conventional drugs include anti-ischemic agents and vascular protective agents, such as nitroglycerin, aspirin, clopidogrel, beta-blockers, and statin (Smith et al., 2011).

In this overview, nine RCTs reviewed the therapeutic effects of PNS on angina pectoris compared PNS + conventional drugs with conventional drugs. It's demonstrated PNS is one effective agents to decrease frequency and duration of angina pectoris. PNS could decrease significantly frequency and duration of angina pectoris. 180 patients of unstable angina were randomly divided into treatment group and control group of, respectively 90 patients. The treatment group added PNS (2 times/d for 4 weeks) on the basis of conventional treatment of angina pectoris. The control group administered conventional treatment of angina pectoris. The results showed that the frequency of unstable angina pectoris, pain intensity and duration were significantly reduced (Kuang et al., 2011).

#### Ischemic changes on ECG

ECG is the other important secondary outcome on evaluating the clinical efficacy against angina pectoris. A total of eight RCTs observed ECG changes with PNS on CAD patients. Positive correlations of PNS and improvements of ECG were reported that ischaemic changes on ECG were attenuated significantly. A RCT divided 100 patients randomly into treatment group and control group. The two groups were given conventional drugs, treatment group plus PNS for 4 weeks. It's elucidated that ECG in the treatment group were better than those in the control group, in company with the curative effect of angina pectoris, FAA, the rate of stopping and the dosage of nitroglycerin (Song et al., 2005). Another RCT reported that with PNS treatment, ECG of 92% CAD patients returned to normal state or rise more than 0.05 Mv of ST segment depression, different significantly with conventional drugs alone (Hou, 2016).

#### Lipid metabolism

Lipid disorder is one of the main risk factors for CAD. A 20% reduction in major coronary events within 5 years was caused by a decrease of 1 mmol/L in LDL level (Baigent et al., 2005). All three RCTs reported effectiveness of PNS on lipids of CAD patients. PNS could decrease significantly TC, TG, LDL. PNS combined with conventional drugs was more effective than conventional drugs alone. In addition, some researches were trying to evaluate PNS alone with atorvastatin. Seventy-one patients with CAD were randomly divided into two groups: PNS group (36 cases) and atorvastatin group (*n* = 35). PNS was given 100 mg orally, while atorvastatin group received atorvastatin 20 mg orally. The results showed that there was no significant difference in the levels of TG, TC, CIMT, and plaque between two groups before and after treatment. There was no significant difference in LDL-C before and after treatment in PNS group, while the LDL-C descended significantly in atorvastatin group. The incidence of abnormal liver function, gastrointestinal reaction, and recurrent cardiovascular events in patients with atorvastatin was significantly higher than PNS (Feng et al., 2016).

In this overview, 15 RCTs observe the effect of PNS as alternative and complementary medicine on secondary outcomes, such as frequency of angina attack, duration of angina attack, ECG and lipid metabolism. And the results illustrated PNS combined with conventional drugs had also significant effects on changing the secondary outcomes.

## Adverse events

A systematic review evaluated the safety of PNS for UA, including six RCTs with 716 participants. Four of the included trials (66.7%) reported adverse effects related to treatment with PNS combined with conventional drugs. The only reported adverse effect was rash at 0.27% (1/363). No severe adverse events were reported (Yang et al., 2013). Another systematic review evaluated an oral *P. notoginseng* preparation for CAD and included 17 randomized clinical trials with 1,747 participants. Nine trials reported adverse events. One trial reported reduced blood pressure and increased heart rates. One trial reported nausea, dizziness, and vomiting. One trial reported erythra, and six trials indicated no adverse events throughout the duration of treatment (Shang et al., 2013).

Focusing on PNS for CAD, nine RCTs reported adverse events in all 17 RCTs. No observable toxicity in liver or kidney function was measured by serum markers. Several RCTs described adverse events that indicated that oral PNS for CAD is not related to adverse reactions (Table 4). Feng et al. (2016) reported 2 cases with elevated transaminase, 1 case with muscle pain, and 1 case with gastrointestinal discomfort in the control group. No obvious adverse reactions were observed in the treatment group (Feng et al., 2016). In the experimental group of Yan et al. (2015), 1 case of subcutaneous hemorrhage and 1 case of positive fecal occult blood occurred. One case of nausea and 1 case of positive fecal occult blood occurred in the control group (Yan et al., 2015). In Yu 2010, 1 case in the experimental group showed a small amount of rash after 3 d of treatment, which was not caused by the treatment (Yu, 2010). No significant difference was observed in the incidence of adverse reactions. Furthermore, PNS was not related to any obvious abnormalities in liver and kidney function.

**Table 4:** The incidence of adverse reactions with PNS for CAD.

**Adverse events**	**The incidence of adverse reactions (experimental)**	**The incidence of adverse reactions (control)**	**References**
Elevated transaminase	0/36	2/35	Feng et al., 2016
Gastrointestinal discomfort	0/36	1/35	Feng et al., 2016
Muscle pain	0/36	1/35	Feng et al., 2016
Subcutaneous hemorrhage	1/28	0/27	Yan et al., 2015
Fecal occult blood positive	1/28	1/27	Yan et al., 2015
Nausea	0/28	1/27	Yan et al., 2015
Rash	1/50	0/50	Yu, 2010
Total	3/214	6/208	

## The mechanisms of PNS on CAD

CAD occurs when atherosclerotic lesions impede blood flow in the coronary artery. The plaque activation causes ischaemia and infarction. Ruptures tend to happen near the thin and easy destroyed fibrous cap where activated immune cells, inflammatory molecules, and proteolytic enzymes are abundant (Santos-Gallego et al., 2014). They can weaken the cap and transform stable plaque to an unstable vulnerable plaque that is more likely to rupture. The pathomechanism of atherosclerosis in CAD is related to inflammation, lipid metabolism, endothelial erosion, coagulation system dysfunction, and apoptosis (Hopkins, 2013). Plaque rupture is the major trigger of CAD (Hansson, 2005), while hypoxia and ischaemia are the pathological manifestations of the disease. In addition, angiogenesis can improve blood flow in the presence of microvascular blockage in CAD.

**Figure 4:**
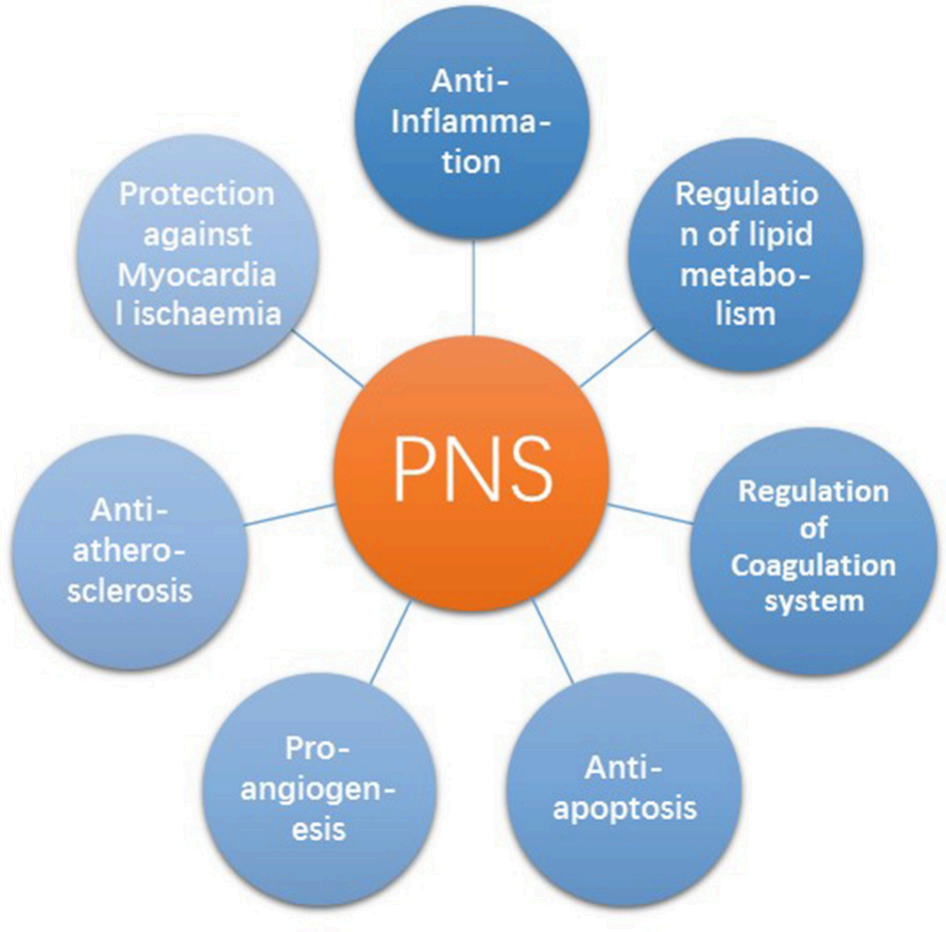
Summary of seven main functions of PNS in CAD.

Saponins are a group of natural compounds in plants and foods. PNS is the most important compound among *P. notoginseng*'s effective components. In the past 10 years, it had received extensive attention in the treatment of CAD at home and abroad. Many studies showed that it had anti-inflammatory, anti-apoptotic, anti-hypoxic, lowering lipids, anti-coagulation, and pro-angiogenesis properties (Table 5, Figure 4).

**Table 5:** Summary of animal and cell experiments of *Panax notoginseng* saponins on CAD.

**Species**	**Experimental model**	**Effects**	**Signaling molecules involved**	**References**
PNS	Human granulocytic HL-60, erythrocytic K562, megakaryocytic CHRF-288, and Meg-01 cell line	Promote proliferation and differentiation	Kinase MEK-1↑, MEK-2↑, ERK-1↑, ERK-2↑, AKT-1↑, AKT-2↑, PI3K↑	Fan et al., 2012
PNS	THP-1 macrophage cells	Reduced secretion of inflammatory factors	LXRalpha↑, ABCA1↑, ABCG1↑, NF-κB↓, IL-6↓, MCP-1↓	Dou et al., 2012
PNS	Apo-E-deficient mice	Inhibit the progression of atherosclerotic lesions via antioxidant/anti-inflammatory biological properties	VCAM-1↓, ICAM-1↓, MCP-1↓, RAGE↓, NF-κB↓, JNK, p38(MAPK)↓, ERK1/2↓	Aronoff et al., 2004
PNS	Peritoneal macrophage cells	Enhanced phagocytosis	COX-2, PGE↓, PGD↑	Yuan et al., 2009
PNS	Haemorrhagic shock rats	Protective to rat haemorrhagic shock model by antioxidative stress and anti-inflammation	ICAM-1↓, SOD↑, MDA↓, endotoxin↓, MPO↓, TNF alpha↓, IL-6↓	Liu H. Z. et al., 2014
NG	Rat washed platelets	Inhibit ADP-induced platelet aggregation	Grb2↑, thrombospondin 1↑, tubulin alpha 6↑, thioredoxin↑, Cu–Zn superoxide dismutase, DJ-1↑, peroxiredoxin 3↑, thioredoxin-like protein 2↑, ribonuclease inhibitor↑, potassium channel subfamily V member 2↑, myosin regulatory light chain 9↑, laminin receptor 1↑	Yao et al., 2008a
Ginsenoside-Rd	Basilar artery smooth muscle cells	Inhibit cell proliferation and reversed basilar artery remodeling	Cytochrome C↑, caspase-9/caspase-3↑, MMP↓, Bcl-2/Bax↓, Cyclosporine A↓	Li et al., 2012
NR1	Human endothelial EA. hy926 cells	Suppress oxLDL-induced inflammatory cytokines production	PPARgamma↑, NF-κB↓, MAPK↓	Su et al., 2016
NR1	Human aortic smooth muscle cells	Inhibits TNF-alpha-induced PAI-1 production	ERK↓, PKB↓	Zhang and Wang, 2006
NR1	H9c2 cardiomyocytes	Reduced cardiomyocyte apoptosis and inflammation	ERalpha↑	Zhong et al., 2015
NR1	Endotoxaemic mice	Protection of cardiac function	ERalpha↑, phospho-Akt↑, phospho-GSK3beta↑, I-κB alpha↑	Sun B. et al., 2013
Ginsenoside Rg1	Hypoxia/reoxygenation cardiomyocytes	Antioxidative effect	ROS↓, T-SOD↑, CAT↑, GSH↑	Zhu et al., 2009
PNS	Foam cells	Decrease cholesterol ester	ABCA1↑	Jia et al., 2010
PNS	CAD rats	Improve lipid metabolism	LPL↑, FABP4↓, CPT-1A↓, cytochrome P450↑, PPARalpha↓, PPARgamma↓, RXRA↓, PGC-1alpha↓	Fan et al., 2012
PNS	Atherosclerosis rats	Regulate the blood lipid profile and anti-inflammation	Integrins↓, IL-18↓, IL-1beta↓, MMP-2↓, MMP-9↓, NF-κB/p65↓, IκBalpha↑	Zhang et al., 2008
PNS	Atherosclerosis rabbit	Regulate the blood lipid profile and anti-inflammation	IL-6↓, CRP↓, MCP-1↓, NF-κB/p65↓	Liu et al., 2010
PNS	apoE(−/−) mice	Prevent the development of atherosclerosis	Ca^2+^ influx↑, SR-A↓	Hall et al., 2005
Ginsenoside-Rd	Macrophage cells	Inhibits ox-LDL-induced foam cell formation	Ca^2+^ influx↑	Hall et al., 2005
PNS	Endothelial cells	Inhibit platelet activation	COX-2, 6-keto-PGF1alpha↑, COX-1↓, TXB2↓	Wang M. M. et al., 2016
PNS	Rats	Inhibit ADP-induced platelet aggregation of platelet rich plasma		Yao et al., 2008b
PNS	Rabbit and human platelet	Anti-platelet aggregation	ERK2↓, p38↓	Qi et al., 2016
PNS, ginsenosides (Rg1, Re, and NR1)	Human plasma	Anticoagulation activity		Li et al., 2013
Ginsenosides, Rg1, Rg2	Rat washed platelets	Enhanced platelet aggregation	Ca^2+^↑, P2Y12 receptors↑	Gao et al., 2014
notoginsenoside Ft1	HEK293 cells	None	Ca^2+^↑, P2Y12 receptors↑, cAMP, phosphorylation of PI3K↑, Akt↑	Gao et al., 2014
NR1	Cultured HUVECs	Activate tissue-type plasminogen	TPA↑, TPA-PAI-1 complexes↑	Zhang et al., 1994
PNS	H9c2 cells	Anti-apoptosis	PI3K↑, p-Akt↑	Li et al., 2014
PNS	Myocardial ischaemia injury rats	Improved cardiac function in rats	p-Akt↑	Wang et al., 2015
PNS	Rat aorta after balloon angioplasty	Inhibit intima hyperplasia by inhibiting VSMCs proliferation	PCNA↓	Wang et al., 2009
PNS	VSMCs	Inhibit VSMCs proliferation and induce VSMCs apoptosis	p53↑, Bax↑, caspase-3↑, Bcl-2↓	Xu et al., 2011
PNS	VSMCs	inhibit VSMCs proliferation	cyclinD1↓, CDK4↓, p21↓, P-ERK1/2↓, MKP-1↑	Zhang et al., 2012
PNS	Human umbilical vein endothelial cells(HUVECs)	Stimulate the proliferation of HUVECs	PI3K↑, Akt↑, eNOS↑	Hong et al., 2009
PNS	Zebrafish	Promote changes in the subintestinal vessels	VEGF-KDR/Flk-1↑	Hong et al., 2009
Notoginsen-oside F1	HUVECs	Pro-angiogenesis, stimulate the proliferation of HUVECs	VEGF-KDR/Flk-1↑, PI3K↑, eNOS↑, Akt↑	Yang et al., 2016
Notoginsen-oside F1	Rat mesenteric arteries	Induce endothelium-dependent relaxation	eNOS↑, ER beta↑, Akt↑, ERK1/2↓	Shen et al., 2014
PNS	Apolipoprotein E-knockout mice	Lower serum lipid levels	CD40↓, MMP-9↓	Liu et al., 2009
PNS	Apolipoprotein E-knockout mice	Reduce the size of atherosclerotic plaque	SDF-1 alpha↑, SCF↑, MMP-9↑, CXCR4↑	Liu et al., 2013
PNS	Zymosan A induced atherosclerosis rats	Inhibit atherogenesis	p-FAK↓, NF-κB↓	Zhang et al., 1994
PNS	Acute myocardial ischaemia in anesthetic dogs	Attenuate the damage of myocardial ischaemia and infarction	ET↓, TXA2↓, MBF↑	Yuan et al., 2011
PNS	Post-myocardial infarction-ventricular rats	Reduce pathological injury of cardiac myocytes in myocardial ischaemia and cardiac muscle	ACE2↑, TNF-alpha↓	Guo et al., 2010
PNS	Rabbits after balloon endothelial denudation (BED)	Promote endothelial, regeneration and reduce extracellular matrix thickening	VEGF↓, MMP-2↓	Liu et al., 2009
PNS	Cardiomyocytes with hypoxia-reoxygenation	Inhibit apoptosis and improve energy metabolism		Gong et al., 2013
NG	Rats of ischaemia-reperfusion (IR)	Cardioprotective effect		Yue et al., 2012
Ginsenoside Rg1, Rb1	Myocardial infarction rats	Improved heart contractility		Deng et al., 2015

### Anti-inflammation

**Figure 5:**
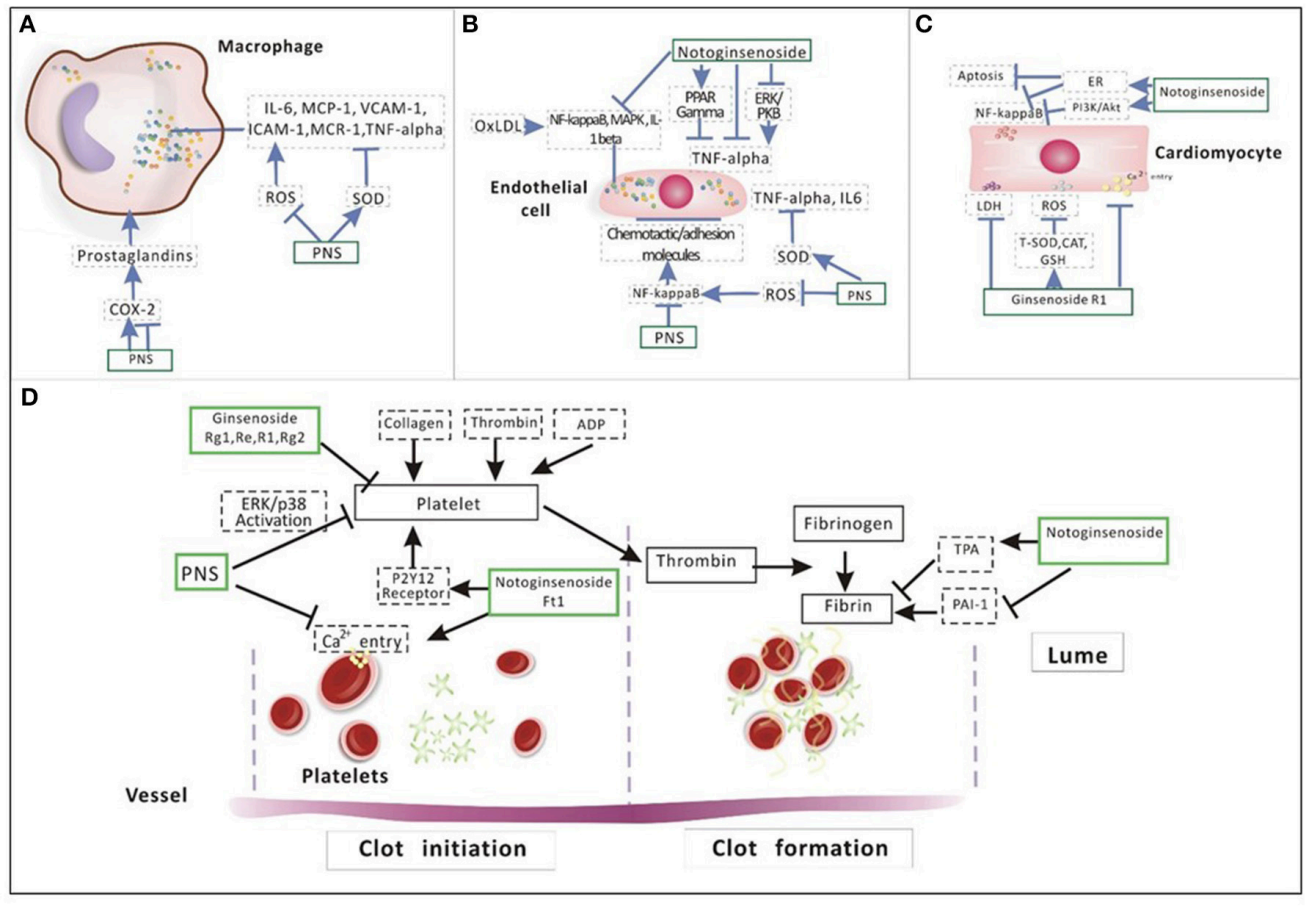
Illustration of the mechanism of PNS on **(A)** macrophage, **(B)** endothelial cell, **(C)** cardiomyocyte, and **(D)** platelet aggregation. In the process of inflammation among macrophages **(A)**, pro-inflammatory factors such as IL-6, MCP-1, VCAM-1, ICAM-1, MCR-1, and TNF-alpha are regulated by ROS and SOD. Prostaglandins produced by COX-2 is negatively related to phagocytosis. PNS can regulate pro-inflammatory factors by inhibiting ROS and promoting SOD. In addition, PNS also inhibit TNA alpha directly. The activated NF-κB regulates the expression of many atherogenic genes, creating a local inflammatory condition and inducing chemotactic factors and adhesion molecules on the surface of ECs **(B)**. PNS can increase SOD activity by decreasing TNF alpha, IL-6 and ROS generation. Notoginsenoside R1 can suppress inflammatory cytokines production by activating PPAR gamma and by suppressing ERK and PKB, inhibiting TNF-alpha. In addition, NR1 can inhibit NF-κB, MAPK, IL-1 beta and reduce cardiomyocyte apoptosis and inflammation through the activation of ER alpha and PI3K/Akt signaling **(C)**. Ginsenoside Rg1 reduced intracellular ROS and LDH and suppressed the intracellular Ca2+ level by increasing the activity of endogenous antioxidants, including T-SOD, CAT and GSH. About **(D)**, NG have an inhibitory effect on platelet aggregation. The effect of PNS in anti-platelet aggregation is related to the suppression of intracellular calcium mobilization and ERK2/p38 activation. Three main ginsenosides (Rg1, Re, and R1) that exist in PNS also showed anti-platelet activity. Ft1 induced dose-dependent platelet aggregation mediated through P2Y12 receptors. NR1 significantly decreased TNF alpha-induced PAI-1.

Inflammation dominates in CAD and atherosclerosis. Immune cells gather in the early atherosclerotic lesions, where effector molecules promote the progress of inflammation which can induce acute coronary syndrome (ACS; Han, 2008). In vulnerable plaques, the main characteristic is that inflammation exists widespread. Several studies showed that different systems of inflammation markers such as C-reactive protein in patients are related to an increased risk of ACS (Crea and Liuzzo, 2013; Mann et al., 2014). And inflammation associated with CAD includes MAPK activation, the role of NF-κB, TNF-α, ROS for signaling, modified lipoprotein on endothelial cells and other cell activation, leukocyte adhesion, formation of foam cells, macrophages (Figure 5A), and vascular smooth muscle cells (VSMCs).

First, NF-κB is critical for the trigger and development of atherosclerosis (Hopkins, 2013). The elevated NF-κB activators such as osteoprotegerin were related to increased mortality, especially of cardiovascular diseases (Venuraju et al., 2010). Activation of NF-κB led to regulation of many atherogenic genes, facilitation of inflammation, induction of chemokines, and adhesion molecules on the surface of endothelial cell (ECs) (Figure 5B). Chemokines and adhesion molecules attracted the monocytes to the location prone to atheroma (Gerits et al., 2007; Marks et al., 2009; Baker et al., 2011). PNS inhibited NF-κB DNA binding activity (Sun X. et al., 2013) and secreting pro-inflammatory factors, interleukin (IL)-6 and MCP-1 in macrophages (Fan et al., 2012). The size of atherosclerotic lesions and the number of macrophages in apolipoprotein E (apoE; −/−) mice were reduced by PNS. In addition, PNS reduced the expression of proinflammatory cytokines VCAM-1, ICAM-1, and MCP-1 with inhibition of NF-κB, JNK, p38 (MAPK), and ERK1/2 activation and RAGE (Dou et al., 2012). Phagocytosis induced the expression of the pro-inflammatory factor COX-2 and the production by COX-2 regulated the functions of macrophage (Aronoff et al., 2004). In the model of inflammation, the expression of COX-2 reached a peak. However, in the later stage COX-2 descended which leads to activation of PPARγ and inhibition of inflammation by NF-κB. Interestingly, PNS suppressed the expression of COX-2 at an early stage with promotion of phagocytosis, while PNS also elevated COX-2 expression at a later stage (Yuan et al., 2009).

Second, oxidation is generally considered as a facilitator or a modulator of inflammatory signaling (Oliveira-Marques et al., 2009), and also a major endothelial-derived hyperpolarizing factor (EDHF) mediator (Capettini et al., 2010). Oxidative stress involves the inflammation of vessels and the progression of atherosclerosis. Excessive reactive oxygen species (ROS) generation has been suggested to up-regulate pro-inflammatory cytokines and adhesion molecules which can result in atherosclerosis initiation consequently (Zhang et al., 2009). ROS facilitated the activation of ROS/Akt/IKK pathways that interact with NF-κB (Qi et al., 2012). In addition, MDA(malondialdehyde) is a product of a free-radical peroxidatic reaction on lipids, and Superoxide dismutase (SOD) is a free-radical scavenger (Van Raamsdonk and Hekimi, 2012). Myeloperoxidase (MPO), a peroxidase enzyme, could accurately predicted the mortality risk in patients with coronary angiography. The improvement of MPO and CRP ameliorated the long-term risk assessment of outcomes in CAD patients (Heslop et al., 2010).

PNS also are considered as free radical-scavengers with antioxidant properties. PNS could impede the development of atherosclerotic lesions through the antioxidant and anti-inflammatory effects (Aronoff et al., 2004). PNS protected a rat haemorrhagic shock model via antioxidative stress and anti-inflammatory pathways. PNS also increased SOD activity, decrease MDA, endotoxin, MPO, TNF alpha, and IL-6 (Liu H. Z. et al., 2014). PNS could reduce oxidative stress and inhibit plaque progression. SOD and glutathione activities were elevated and ROS generation is impaired in apoE(−/−) mice treated with PNS (Aronoff et al., 2004).

Treatment with Notoginsengnosides (NG) could decrease the ROS level in platelets (Shang et al., 2013). Ginsenoside-Rd significantly promoted H_2_O_2_-induced cell apoptosis with a concentration-dependent manner (Li et al., 2012). NR1 with the effect of phytoestrogen, was illustrated as a component with anti-inflammatory, antioxidative and anti-apoptotic properties. NR1 can restrict oxidized low-density lipoprotein (ox-LDL)-induced inflammatory cytokines including NF-κB, MAPK, TNF-alpha, and IL-1 beta (Su et al., 2016). It also inhibits PAI-1 overexpression by TNF-alpha in human aortic smooth muscle cells (HASMCs) and the ERK/PKB pathways (Zhang and Wang, 2006). NR1 can protect the heart from septic shock, probably through the activation of ER alpha and PI3K/Akt pathway. This mechanism blocked NF-κB activation and attenuated inflammation and apoptosis in the myocardium (Sun B. et al., 2013; Zhong et al., 2015). In addition, the pretreatment with ginsenoside Rg1 decreased the release of lactate dehydrogenase(LDH) and increased cell viability dose-dependently. Ginsenoside Rg1 suppressed ROS and Ca^2+^ level intracellularly by raising the activity of endogenous antioxidants as T-SOD, CAT, GSH (Zhu et al., 2009).

### Regulation of lipid metabolism

Lipoprotein disorder is one of the main risk factors of CAD. A meta-analysis of 14 randomized trials showed that a decrease of 1 mmol/L in plasma LDL levels generates a 20% reduction in major coronary events including coronary revascularization and stroke within 5 years (Baigent et al., 2005). CAD is closely related to lipid metabolic disorders, specifically increased triglycerides (TG), low-density lipoprotein cholesterol (LDL-C), ox-LDL, and total cholesterol (TC; Labreuche et al., 2010; Nyyssonen et al., 2012). PNS can reduce remarkably the level of cholesterol ester in foam cells by up-regulation of ABCA1. This bioactivity may be related to the special chemical structures of PNS that are like the natural agonist of liver X receptor alpha (LXR alpha; Jia et al., 2010). LXRα as a key regulator of macrophage function, controls transcriptional programmes involved in lipid metabolism and inflammation (Christoffolete et al., 2010). PNS could regulate lipids by activation of the LXR alpha gene promoter which increased ABCA1 and ABCG1 subsequently and suppressed NF-κB DNA binding activity (Fan et al., 2012).

PNS could markedly reduce TC, TG, and LDL-C (Zhang et al., 2008) and increase high-density lipoprotein cholesterol (HDL-C) significantly (Liu et al., 2010). CPT-1A is a key enzyme in the process of fatty acid oxidation. While the fatty acid transporter protein 4 (FATP4) is associated with long and very long chain fatty acid uptake and promoting synthesis of acyl-CoA (Hall et al., 2005). Fatty acid binding protein 4 (FABP4) and CPT-1A, were downregulated in ischemic zone of the heart. PNS could regulate lipid metabolism by increasing the expression of FABP4 and CPT-1A (Wang Q. et al., 2016).

Lipid metabolic disorder can be caused by inflammation and can exasperate the inflammation (Hotamisligil, 2006). Dyslipidemia and inflammation accelerate each other to form a detrimental cycle. Regulation of lipid metabolism disorders is conducive to inflammation alleviation and anti-inflammatory effects benefits the maintenance of balanced lipid metabolism. PNS could regulate lipid metabolism. Meanwhile, PNS decreased significantly the expressions of some inflammatory cytokines including integrins, IL-18, IL-1 beta, and matrix metalloproteinases 2 (MMP2) and 9 (Zhang et al., 2008).

In conclusion, CAD is closely related to lipid metabolic disorders, specifically including increased TG, LDL-C, ox-LDL, and TC. PNS could depress the level of TC by elevating LXR alpha, ABCA1, and ABCG1 and reducing NF-κB. In addition, PNS can regulate lipid metabolism by inhibiting LPL and increasing FABP4 and CPT-1A. Furthermore, lipidosis is closely related to inflammation, which PNS have diverse effects on.

### Regulation of coagulation system

In CAD, antiplatelet therapy has become an important treatment according to several important guidelines (Chew et al., 2016; Levine et al., 2016). Near wound, platelets are recruited to restore endothelial integrity to initiate thrombus formation (Nording et al., 2015). Thrombosis is associated with platelet aggregation. Thromboxane A2 (TXA2), derived from platelets, induces powerfully release and aggregation of platelets.

PNS inhibited platelet activation by multiple ingredients and pathways. PNS could decrease platelet activation, inhibit adhesion and aggregation of platelet, prevent thrombosis, and improve microcirculation (Wang et al., 2004). PNS protected ECs from injury by suppressing platelet adhesion, in which PNS was superior to aspirin. The underlying mechanism is related to the COX pathway in both ECs and platelets (Figures 5B,D; Wang M. M. et al., 2016). NG could suppress platelet aggregation *in vitro*. Furthermore, *in vivo* NG could also significantly inhibit platelet aggregation of platelet rich plasma (PRP; Yao et al., 2008b). The effect of PNS in anti-platelet aggregation is associated with inhibition of intracellular calcium mobilization and activation of ERK2/p38. Three main ginsenosides (Rg1, Re, and NR1) existing in PNS also demonstrated anti-platelet activity, but their combination did not exhibit any synergistic effect on rabbit platelet aggregation (Qi et al., 2016). Rg1 and Rg2 can significantly prolong the clotting time. Compared with Rg1, Rg2 showed a stronger anticoagulant effect (Li et al., 2013).

However, in PNS, notoginsenoside Ft1 as the potent procoagulant component induced platelet aggregation dose-dependently. The P2Y12 receptor serves as a crucial regulator of haemostasis and thrombosis on the platelet. When conditioned by ADP, the P2Y12 receptor activated a series of downstream events that result in platelet aggregation, shape change, dense granule secretion (Dorsam and Kunapuli, 2004; Gao et al., 2014). Ft1 deceased plasma coagulation indexes and tail bleeding time and increased thrombogenesis and cytosolic Ca^2+^ accumulation.

Fibrinolysis is part of the coagulation cascade, which is adjusted by plasminogen activator (PA) and PA inhibitor (PAI-1). Abnormal fibrinolysis and high plasma concentrations of PAI-1 are related to an increased risk of CAD (McBane et al., 2010). When human umbilical vein endothelial cells (HUVECs) were conditioned with purified NR1, tissue-type PA (TPA) synthesis increase in a dose- (0.01–100 mg of NR1/mL) and time-dependent manner. NR1 significantly decreased PAI-1 mRNA, protein and secretion in HASMCs in a dose-dependent manner (Zhang and Wang, 2006). TPA activity and TPA-PAI-1 complexes reached greater than two-fold and threefold maximal stimulation, respectively NR1. In contrast, NR1 induced five-fold decrease in PAI-1 activity (Zhang et al., 1994).

### Anti-apoptosis

Myocardial ischaemia can lead to widespread cell apoptosis (Ohno et al., 1998). PI3K/Akt pathway is an important regulator including proliferation, apoptosis and nitric oxide (NO) synthesis (Blanes et al., 2007). PI3K also strengthens the oxidative capacity of cardiac fatty acid. The PI3K signaling cascade diminishes myocardial damage by ischaemia via recruiting several endogenous cardioprotective pathways (Hausenloy et al., 2005).

PNS could protect myocardial cells from apoptosis induced by ischaemia both *in vitro* and *in vivo* by activating the PI3K/Akt signaling pathway (Tello-Montoliu et al., 2006). PNS significantly up-regulated p-Akt in H9c2 cells and ischaemic myocardial tissues. PNS attenuated cell apoptosis via chromatin concentration and condensation by up-regulating the antioxidative abilities of SOD and MDA (Li et al., 2014). PNS also improved cardiac function in the left ventricular ejection fractions (EF) of rats (Chen et al., 2011; Wang et al., 2015).

The pathological proliferation of VSMCs is a crucial factor involved in the pathogenesis of atherosclerosis, associated with inflammation, apoptosis, and matrix alterations (Zakar and Ken, 2003). PNS suppressed proliferation and induced apoptosis in VSMCs (Wang et al., 2009) by up-regulating p53, Bax, and caspase-3 and down-regulating Bcl-2 (Xu et al., 2011). In addition, both atorvastatin and PNS have been observed to suppress VSMC proliferation by inhibiting the activation of the ERK signaling pathway (Zhang et al., 2012).

### Pro-angiogenesis

Angiogenesis is the stimulation of the endothelium to shape new blood vessels, which is implicated in the pathophysiology of CAD (Tello-Montoliu et al., 2006). In CAD, inflammation related to atherogenesis contributes to the interaction of angiogenic factors (Patel et al., 2006), which lead to vascular repair (Chong et al., 2004). Various angiogenic growth factors and progenitor cells can promote the formation of new blood vessels (Mitsos et al., 2012). Angiogenesis is a potential treatment in many physiological processes such as MI, chronic cardiac ischaemia, and stroke (Giacca and Zacchigna, 2012).

PNS could enhance angiogenesis and the proangiogenic effects including the VEGF-KDR/Flk-1 and PI3K-Akt-eNOS signaling pathways *in vivo* and *in vitro* (Hong et al., 2009). NR1, similar to Rg1 and Re, had been shown to have pro-angiogenic effect, possibly by activation of the VEGF-KDR/Flk-1 and PI3K-Akt-eNOS signaling pathways *in vivo* and *in vitro* (Yang et al., 2016).

Ft1 can stimulate angiogenesis. Ft1 led to proliferation, migration and tube formation in HUVECs by activation of the PI3K/Akt and ERK1/2 pathways in rat mesenteric arteries. This leads to the phosphorylation of eNOS and release of NO, which triggers soluble guanylyl cyclase in the VSMCs (Shen et al., 2014).

### Anti-atherosclerosis

Atherosclerosis is the pathological basis of CAD. Furthermore, the development of chronic atherosclerosis to form thrombosis is the pathogenesis of ACS (Mann et al., 2014). ApoE is a ligand for cleansing receptors of chylomicrons and very low density lipoprotein residues. The lack of apoE can lead to the accumulation of cholesterol-rich residues in plasma, and long-time accumulation can generate atherosclerosis (Heeren et al., 2006) with hypercholesterolemia and spontaneous arterial lesions (Meir and Leitersdorf, 2004). PNS was able to decrease lipids, ox-LDL in serum and the expressions of CD40 and MMP-9 in apoE(−/−) mice (Liu et al., 2009). Meanwhile, PNS lessened the size of atherosclerotic plaques, partly by progenitor cell mobilization. PNS also augmented endothelialization and reduced the VSMC content of the lesions (Liu et al., 2013).

A high-fat diet together with Zymosan (Zym) induces atherogenesis in rats. PNS reduced the levels of TC, TG, LDL-C, IL-6, and C-reactive protein and increased the HDL-C level significantly in serum of atherosclerosis rabbits by inhibiting FAK phosphorylation, integrins expression and NF-κB translocation (Yuan et al., 2011). And PNS significantly down-regulated MCP-1 and NF-κB/p65 with 8 weeks of treatment (Liu et al., 2010).

Ginsenoside Rd, isolated from PNS, is a voltage dependent Ca^2+^ channel blocker. Ginsenoside Rd decreased remarkably the size of atherosclerotic plaque and ox-LDL of macrophage in the apoE(−/−) rats. *In vitro*, ginsenoside Rd suppressed the formation of foam cells induced by ox-LDL and cholesterol accumulation in macrophages (Li et al., 2011).

### Protection against myocardial ischaemia

PNS exerted a certain degree of improvement on myocardial ischaemia (Tang et al., 2010). In fact, the earliest study to demonstrate the effects of *P. notoginseng* on CAD was a study published in 1972, in which the oral administration of *P. notoginseng* reduced the dosage requirement of nitroglycerin and improved ECG in clinic. And *P. notoginseng* significantly increased coronary blood flow in dogs (Department of Cardiology in Second Affiliated Hospital of Wuhan Medical College, 1972). PNS was shown to obviously alleviate the degree of myocardial ischaemia and narrow the ischaemic area subjected to myocardial ischaemia and infarction (Fu et al., 2006). PNS could enhance left ventricular systolic and diastolic functions, decrease peripheral resistance, and improve the cardiac function of rats with post-myocardial infarction left ventricular remodeling (Guo et al., 2009). The endothelium was denudated completely after balloon endothelial denudation (BED). PNS could sustaine anti-restenotic effects after BED injury. PNS promoted endothelial regeneration and reduced ECM thickening (Chen et al., 2004). *In vitro*, PNS exhibited an anti-apoptotic effect both in oxygen-deprived H9c2 cells and in ischaemic myocardial tissues (Chen et al., 2011). In addition, PNS could decrease the pathological injury to cardiac myocytes with ischaemia and improve ventricular remodeling (Guo et al., 2010).

The PPAR family, a series of transcription factors, regulates cardiac energy metabolism and impacts metabolism of cardiac fatty acid and glucose (Madrazo and Kelly, 2008). PGC-1α is a transcriptional coactivator of the PPARs and a critical factor in myocardial metabolism (Huss et al., 2004). In ischaemic rats, transcriptional factors were downregulated such as PPARs, RXRA, and PGC-1 alpha (Wang Q. et al., 2016). PNS could up-regulate expressions of these factors.

In addition, salvianolic acids' compatibility with PNS could protect cardiomyocytes (Figure 5C) during hypoxia and reoxygenation injury by inhibiting apoptosis and improving energy metabolism compared to any single drug (Gong et al., 2013). In the model of ischaemia/reperfusion, salvianolic acids (SA), NG, and a combination of SA and NG exhibited the cardioprotective effects. SA and NG displayed both similarities and differences in pathways such as energy metabolism, lipid metabolism, cell proliferation, and apoptosis (Yue et al., 2012). The combination of SalB and Rg1, instead of SalB and Rb1, advanced cardiac contractility in rats with MI (Deng et al., 2015).

## Discussion

### Summary of current evidence

**Figure 6:**
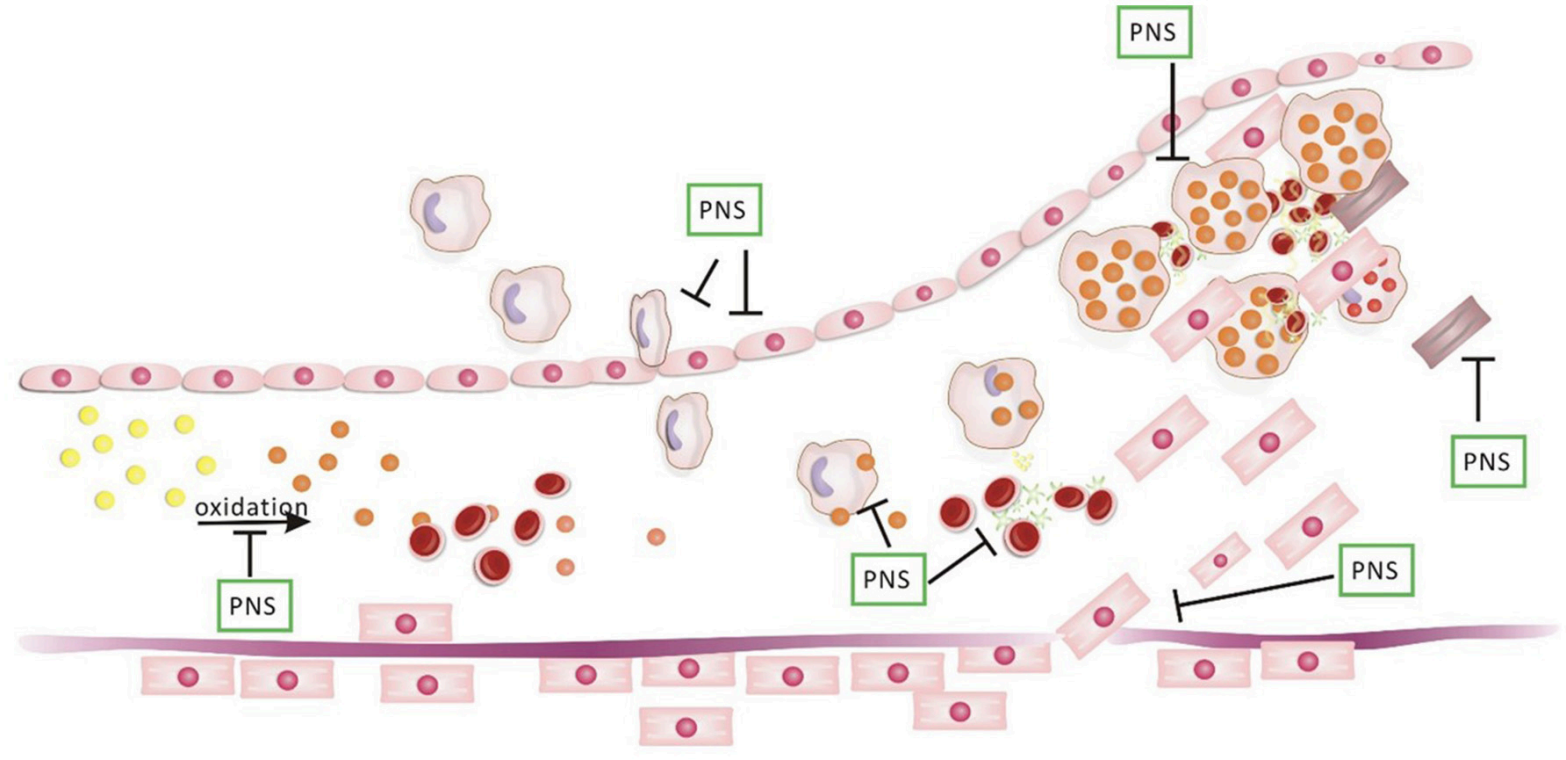
PNS on the evolution of atherosclerotic plaque. In the evolution of atherosclerosis plaque, PNS has effects on the oxidation of LDL, the accumulation of lipoprotein, chemoattractant cytokines related to macrophages, modified lipoprotein particles, platelet aggregation, the migration of smooth muscle cells (SMCs), apoptosis of SMCs and the development of foam cells.

In the past two decades, a breakthrough has been achieved in the pharmacology of PNS. The knowledge of PNS functions offers a new opportunity for the prevention and treatment of CAD. PNS has been observed to have multiple positive effects in the key processes of CAD, including anti-inflammation, the regulation of lipid metabolism and the coagulation system, anti-apoptosis, pro-angiogenesis, anti-atherosclerosis, and anti-myocardial ischaemia (Figure 6). Several RCTs have shown that the long-term use of PNS can effectively reduce the end-point of CAD (Meng et al., 2013; Yan et al., 2015). In addition, many RCTs found that PNS can also significantly improve performance on the ECG and reduce the frequency and the duration of angina attacks, greatly regulating the lipids (Song et al., 2005; Han, 2008; Liu et al., 2008; Du and Chen, 2009; Zhou and Bai, 2009; Wei, 2010; Yu, 2010; Kuang et al., 2011; Wan, 2011; Meng et al., 2013; Teng, 2014; Zhang, 2014; Zheng, 2014; Yan et al., 2015; Hou, 2016).

The function of PNS on platelet aggregation resembles aspirin. For the patients with aspirin resistance (Arachidonic acid inhibitory rate <50%) and clopidogrel resistance (Adenosine diphosphate inhibition rate <30%), Ticagreloran, an oral reversibly binding P2Y12 inhibitor, is commonly used alternative drug (Nylander and Schulz, 2016). However, Ticagrelor is unsuitable for patients with bleeding tendency, meanwhile it means a big financial burden on patients or the government. For those patients, PNS is recommend for anti-platelet aggregation. Several studies *in vitro* and *in vivo* PNS may inhibit the activation of platelet through multiple components and multiple pathways. In a RCT, PNS alone is illustrated to decrease platelet aggregation and less adverse events (Yu, 2010).

Statins as a main drug by many guidelines have shown good effects in the primary and secondary prevention of CAD (Harris et al., 2015; Jellinger et al., 2017). However, statins have side effects including elevated liver transaminase, myopathy, myalgia, myositis, and even rhabdomyolysis. PNS could regulate lipid metabolism, not so strongly as statins, but with a higher safety. Meanwhile PNS has the roles of anti-inflammation and anti-platelet aggregation. So, for patients with liver damage, or the elderly without high TC, TG, LDL, we recommend PNS for lipid-lowering and anti-inflammation.

In addition, nitroglycerin is one of the oldest of cardiovascular drugs in clinics. Nitroglycerin exerts anti-ischemic effect mainly by expanding capacity vein, reducing preload and releasing coronary artery spasm. PNS could reduce myocardial ischemia and relieve angina pectoris. In clinic PNS has similar effects and different mechanisms with nitroglycerin. PNS was recommended when patients can't tolerate the side effects of nitroglycerin such as headache, dizziness, tachycardia. Patients with nitrate resistance were also recommended to administrate PNS.

Inflammation runs through the initiation, formation and onset of CAD. The widespread presence of inflammation generates the major feature of vulnerable plaques (Crea and Liuzzo, 2013). PNS exerts anti-inflammation by several signaling pathways. PNS can regulate pro-inflammatory factors by inhibiting ROS, TNA alpha, NF-κB, and promoting SOD. Interestingly, PNS has dual functions on COX-2 at different periods. An individual PNS, NR1 can suppress inflammatory cytokines production by activating PPAR gamma and by suppressing ERK and PKB, inhibiting TNF-alpha *in vitro* and *in vivo*. Inflammation plays an important role in the whole process of CAD, PNS with less side effects could be administered for a long time. Meanwhile, long-term application of PNS could reduce the end point of CAD.

Different with western medicines, TCM acts on several targets to play a variety of roles on the mechanisms of the disease. PNS has effects simultaneously on anti-inflammation, the regulation of lipid metabolism and the coagulation system, anti-apoptosis, pro-angiogenesis, anti-atherosclerosis, and anti-myocardial ischaemia. In the entire pathological process of CAD, at different pathological stages, PNS can effectively reduce the occurrence and the development of CAD. In several RCTs, PNS effectively reduce the end-point of CAD, greatly regulate the lipids, improve performance on the ECG and reduce the frequency and the duration of angina attacks. Thus, PNS is a potential agent against CAD.

Recently, the medical community has gradually assigned importance to the primary prevention of CAD with PNS due to their unique advantages. In primary prevention, PNS can regulate lipid metabolism and hypertension (Pan et al., 2012) and inhibit platelet aggregation. Aspirin has side effects such as gastrointestinal reactions and statins can cause liver injury. Especially in preventive treatment, long-term administration will increase the rate of side effects. And PNS appear to be safer. PNS have multiple targets, a wide range of therapeutic effects and high safety. Therefore, we believe that it has great potential in the treatment of CAD.

### Limitations and perspectives

Currently, increasing research is focusing on individual PNS. However, these studies are still rare compared to those on total PNS. Individual PNS could have contradictory functions, especially on platelets. Four main ginsenosides (Rg1, Re, NR1, and Rg2) that exist in PNS also showed anti-platelet and anticoagulation activity. Both Rg1 and Rg2 could significantly extend blood clotting time. However, notoginsenoside Ft1 was procoagulant and induced dose-dependent platelet aggregation. Therefore, PNS could be further separated in order to thoroughly investigate the function of *P. notoginseng*. In addition, the mechanism of PNS in CAD is complicated, so the work of individual PNS multi-target networks will further raise the potential of *P. notoginseng* for the effective treatment of CAD.

In addition, we need to further improve the drug purity and screen concentrations to reveal and enhance the medicinal value of PNS as an individual lipid-lowering drug or an antiplatelet agglutination drug. We have found some research that focuses on comparing PNS and aspirin or PNS and statins. The inhibitory effect of PNS on platelet activation was similar to aspirin, but the inhibitory effect of PNS on platelet adhesion to ECs was superior to aspirin (Wang M. M. et al., 2016). In a RCT OF PNS group, aspirin group and PNS plus aspirin group, the results showed decreased D-dimer, platelet aggregation time, increased international standardization ratio of prothrombin time and prolonged prothrombin time in three groups. Compared with PNS, aspirin was more effective than PNS in improving platelet aggregation (Yan et al., 2015). The purity and concentrate increase probably generate stronger effects on CAD.

However, the medical technology and the related animal experiments and RCTs are limited. Moreover, only one 1-year RCT has reported the effect of PNS on the end-point of CAD. However, we hope that some multi-center, large-sample RCTs will provide high-level evidence for the effectiveness of PNS in CAD.

## Conclusion

PNS have multiple positive effects in the key processes of CAD, including anti-inflammation, the regulation of lipid metabolism and the coagulation system, anti-apoptosis, pro-angiogenesis, anti-atherosclerosis, and anti-myocardial ischaemia. Long-term use of PNS can effectively reduce the end point of CAD and improve angina pectoris, ECG and lipid metabolism which illustrates that PNS is potential agent on CAD. However, more high-level RCTs are expected to provide evidence for the efficacy of PNS in CAD.

## Author contributions

JW and LD designed the work of review; LD, XX, and JH reviewed the literature available on this topic and wrote the paper; XX, YL, and JL contributed in the scientific writing of the manuscript; JW and XX revised the manuscript.

### Conflict of interest statement

The authors declare that the research was conducted in the absence of any commercial or financial relationships that could be construed as a potential conflict of interest.

## Footnotes

^1^ChemFaces http://www.chemfaces.com/
